# Concerns Related to the COVID-19 in Adult Norwegians during the First Outbreak in 2020: A Qualitative Approach

**DOI:** 10.3390/ijerph18084312

**Published:** 2021-04-19

**Authors:** Laila Skogstad, Inger Schou-Bredal, Tore Bonsaksen, Trond Heir, Øivind Ekeberg, Tine Grimholt

**Affiliations:** 1 Department of Research, Sunnaas Rehabilitation Hospital HF, 1453 Bjørnemyr, Norway; 2 Department of Nursing and Health Promotion, Faculty of Health Sciences, Oslo Metropolitan University, 0130 Oslo, Norway; 3 Faculty of Medicine, University of Oslo, 0316 Oslo, Norway; inger.schou-bredal@medisin.uio.no; 4 Department of Health and Nursing Sciences, Faculty of Social and Health Sciences, Inland Norway University of Applied Sciences, 2418 Elverum, Norway; tore.bonsaksen@inn.no; 5 Faculty of Health Studies, VID Specialized University, 4306 Sandnes, Norway; 6 Norwegian Center for Violence and Traumatic Stress Studies, 0409 Oslo, Norway; trond.heir@medisin.uio.no; 7 Institute of Clinical Medicine, University of Oslo, 0316 Oslo, Norway; 8 Psychosomatic and CI Psychiatry, Division of Mental Health and Addiction, Oslo University Hospital, 0424 Oslo, Norway; oeekeber@online.no; 9 Faculty of Health Studies, VID Specialized University, 0370 Oslo, Norway; Tine.Grimholt@vid.no; 10 Department of Acute Medicine, Oslo University Hospital, 0424 Oslo, Norway

**Keywords:** concerns, coronavirus, COVID-19, decision-making, population, SARS-CoV-2, qualitative

## Abstract

Concerns related to the first outbreak of the COVID-19 pandemic in the Norwegian population are studied in a cross-sectional web-survey conducted between 8 April and 20 May 2020. The qualitative thematic analysis of the open-ended question “Do you have other concerns related to the pandemic?”, followed a six-step process. Concerns from 1491 informants were analyzed, 34% of women and 30% of men (p = 0.05) provided concerns. Respondents with higher educational level reported concerns more often (86% vs. 83%, p = 0.022). The qualitative analysis revealed five themes—society, health, social activities, personal economy and duration—and 13 sub-themes, mostly related to the themes “society” and “health” (724 and 704, respectively). Empathy for others was prominent, for society (nationally and globally), but also concerns related to infecting others and family members at risk for developing serious illness if infected. The responses to the open-ended question yielded additional information, beyond the information obtained from questions with pre-categorized response options, especially related to concerns about society and health. Themes arising from the qualitative analysis shed light on what are important concerns for people during the pandemic and this may serve as targeted measures for the authorities.

## 1. Introduction

On 12 March 2020 a lockdown was imposed in Norway, at an early stage compared with other countries [1]. “Social distancing” became the main policy for public behavior [2,3]. Encouragement to restrict physical contact with persons outside one’s own household and even to stay at home, was expressed. Kindergartens, schools and universities were closed, but digital home teaching was introduced for schools/universities. Non-vital businesses, e.g., cultural events and travels, were cancelled, leading to financial problems and many became unemployed or made redundant [4]. Strict measures such as these and fear of infection, forcing a drastic change in people’s habits and routines, might be perceived as a threat.

**Figure 1:**
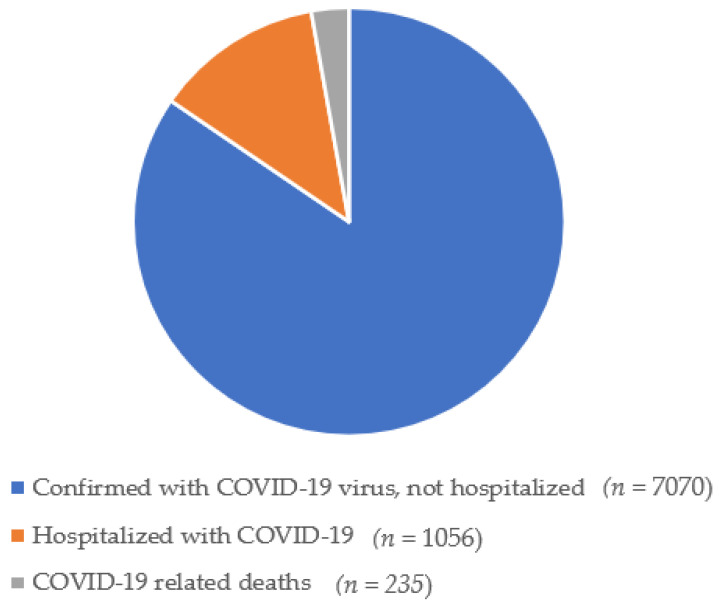
COVID-19 in Norway from March–June 2020.

In June 2020, approximately 5.4 million people were registered as Norwegian citizens; of those, 235.706 had been tested for SARS-CoV-2 [5]. A small proportion was confirmed with COVID-19, as shown in Figure 1.

A clear and transparent communication from the authorities has been recommended to establish trust and high degree of compliance [6,7]. The authorities’ call to join a collective effort seems to have created a strong team spirit in Norway [2], since few were confirmed with COVID-19 virus, admitted to hospital or registered as dead due to COVID-19 during the first outbreak. However, the pandemic still may have led to concerns.

Population-based studies on COVID-19 have approached concerns in different ways. The risk perception of COVID-19 was uniformly high in ten countries across the world, with England at the top [8]. People who experienced personal and direct experience with the virus perceived higher risk and socio-cultural factors and knowledge explained some of the variance in concerns [8]. The burden of stressors causing health and economic worries was found across EU in a study addressing containment policies, worries and trust in sources of information [6]. The burden was lower in the northern relative to the southern parts of Europe and found to be prominent also in households not directly affected by COVID-19.

Nelson et al. [9] found that 67% of 8959 participants in the US reported to be very or extremely concerned about COVID-19. Getting sick and not receiving medical care were among top 10 concerns. About 15% reported reduced wages or work hours and 1.5% reported having lost their jobs. People in areas with more infected individuals in the US reported higher degree of fear, worry and anxiety [10]. According to Sabat et al. [6], losing a close person, becoming unemployed, health system becoming overloaded, closed school, small companies running out of business, recession and society getting more egoistic have been reported as COVID-19 concerns.

In the US and Canada, Taylor et al. [11] identified that COVID-19 stress reactions were more complex than fear of being infected. COVID stress syndrome reactions also included perceptions of danger and being infected from surfaces; socioeconomic costs; fear of foreigners spreading the virus; traumatic stress symptoms associated with direct or vicarious exposure to COVID-19 and compulsive checking and reassurance seeking. In a published paper from the present CORONAPOP study, the prevalence rates of symptom-defined PTSD were 12.5% for men and 19.5% for women [12]. This was a higher compared to Norwegians in 2015, where 3.8% of men and 8.5% of women reported symptom-defined PTSD [13]. Women seem to be more sensitive to concerns and stress compared to men [10,11,12,13,14,15].

A meta-analysis on anxiety and depression during the pandemic included 60 studies [16]. Most were general population samples (40.2%). In addition, healthcare providers (23.8%), students (16.4%) and patients (8.9%) were included. The global prevalence of anxiety was 21.3% and depression was 24%. Asia had lower levels compared to other parts of the world. Hoffart et al. [17] found that loneliness in the Norwegian population was associated with depression and anxiety, but with a small effect size. Rumination and worry in general were associated with more loneliness.

Resilience and personality traits may serve as protective factors. Pagnini et al. [18] found an association between worries and emotional stability in people living in quarantine area of Italy during the first week of the pandemic. They suggested that emotionally stable (resilient) people experienced less concerns and fear. This was in line with findings in a study by Flesia et al. [15] where stable traits such as self-control, emotional stability and internal locus of control were protective factors. We found that optimists, e.g., emotionally stable, in general were less worried about COVID-19 relative to pessimists and they showed better global health [19]. In addition, more pessimists reported being at risk for COVID-19 even in the absence of known risk factors.

Lockdown of the society has impact on symptoms of psychological distress and concerns, but few in-depth interviews about concerns have been published. We found three qualitative studies. Two-thirds of 40 Spanish informants [14] reported an increase in worrying about suffering from or contracting an illness (COVID-19 or other) relative to before the pandemic. Fear of losing loved ones increased in 76% and psychological distress such as sadness/depression, anxiety, anger and worrying were identified. In a Danish study, Clotworthy et al. [20] found few alarming changes regarding worry, quality of life and social isolation, but some expressed worries related to the health of loved ones and economic consequences. In a master thesis from Norway [21], empathy towards others was prominent, but also an increased attention to the importance of family and friends and concerns about socioeconomic consequences. Some informants perceived the pandemic as exaggerated.

In addition, we found two qualitative content analyses on comments, one from Singapore [7] and one from Uruguay [22]. Shorey et al. [7] identified worries about what would happen to the affected businesses, virus transmitted through tourists and contracting the virus from colleagues and/or at public transportation. In Uruguay, Ares et al. [22] identified worries about the health of family members, employment and the economic situation for oneself and the country.

When we performed the survey, as far as we know, no studies had been published using open-ended or in-depth questions about concerns. The few studies published during 2020/2021 are presented above.

Most studies of concerns are quantitative surveys with pre-categorized response options, but a fixed list is probably not sufficient to cover all concerns people may have. Thus, the objective of the present study was to explore the content of concerns in the Norwegian adult population.

## 2. Materials and Methods

### 2.1. Design

CORONAPOP is a cross-sectional population-based survey, which includes open-ended questions. It was conducted between 8 April and 20 May 2020. A web-link was open to all citizens using different approaches: Oslo University Hospital, Sunnaas Rehabilitation Hospital, University of Oslo and Oslo Metropolitan University hosted and disseminated the link. Further, a snowball sampling strategy was utilized by sharing the link to the survey on social media platforms, such as Facebook, Twitter, LinkedIn and Instagram. In addition, national and local newspapers featured the study with an online link to the electronic questionnaire.

The inclusion criteria were 18 years or older, Norwegian was the only language option at the web site and there were no exclusion criteria. An open web-link provide no information on a possible number of participants and, thus, there is no information on response rate.

The cross-sectional survey consisted of 4527 respondents, with 85% women, mostly (90%) between 18 and 59 years old and living in a city with more than 100,000 inhabitants (46.3%). More than half were married (60%) or had a boy-/ girlfriend (7%) and 34.2% were living with a child/adolescent. Most of the respondents reported higher education (84.3%) and 87.8% were part of the working force or studying before the COVID-19 pandemic with a decrease to 81.0% during the first wave of the pandemic.

The present paper contains a qualitative analysis of comments provided in the cross-sectional population-based survey to the open-ended question: “Do you have other concerns related to the pandemic? If so, please elaborate on your concerns”.

The qualitative approach is based on an explorative attitude regarding what concerns might turn out to be common among the respondents. We expected to see concerns related to fear of being infected or transferring the virus to relatives or friends and economic concerns, but the open-ended question allowed for exploring a variety of other concerns.

### 2.2. Participants

#### Sample Characteristics

To be included in the qualitative part of the study, the respondent had to make a comment to the open-ended question.

“Do you have other concerns related to the pandemic? If so, please elaborate on your concerns”.

Of the 4527 respondents, 1491 (32.9%) presented 1961 concerns in their response to the open-ended question, 33.5% women and 29.6% men, more often those between 18–39 years of age, made comments.

### 2.3. Measures

A qualitative analysis of the open-ended question related to concerns was the focus in the present paper, but some sociodemographic variables and results from pre-categorized response options about concerns for the total population are presented. Most of the questions in CORONAPOP are identical to the questions used in a population study (NORPOP) performed in 2015 [23], but they were adjusted to the pandemic situation.

#### 2.3.1. Sociodemographic Variables for the Total Population

Data were collected for age groups, gender, highest completed educational level (high school or lower versus higher education) and current and pre-COVID employment status (employed or in education: yes). In addition, relationship status (married, boy/girlfriend, widow, divorced, single: yes), living situation (alone, with parents, spouse, person > 18 years, children < 18 years) and place of residence (<200 inhabitants, 200–19,999 inhabitants, 20,000–99,999 inhabitants, 100,000 inhabitants or more). The question “Do you have friends that will provide help when you need it?” with response options yes or no, assessed social support.

#### 2.3.2. Data Regarding Infection

Questions regarding being infected by COVID-19 were assessed: have you been infected with the coronavirus; been quarantined or in isolation; having risk factors for developing complications if infected by the coronavirus: for all, the response alternatives were yes/no.

#### 2.3.3. Concerns Related to COVID-19

We provided five pre-categorized questions with response alternatives yes/no: whether they in relation to COVID-19 had concerns related to personal economy; economic loss; concern for family/friends; being at risk for complications if infected; or general concern for the pandemic.

An open-ended question was then applied: “Do you have other concerns related to the pandemic?” If so, please elaborate on your concerns.

### 2.4. Statistical Analyses

Proportions (%) and frequencies were calculated for the categorical variables and age-groups, gender and educational level were cross tabulated with response or non-response to the open-ended question about other concerns (Pearson Chi-Square). IBM SPSS Statistics (version 24, IBM Corp., Armonk, NY, USA) was used for statistical analyses. Results from the quantitative questions about concerns will be presented in a separate paper [24].

### 2.5. Thematic Analysis

The thematic analysis of the qualitative comments followed a six-step process developed by Virginia Braun and Victoria Clarke: “Familiarization”; “Coding”; “Generating themes”; “Reviewing themes”; “Defining and naming themes”; “Writing up” [25]. We used an inductive approach to allow the data to determine the themes and a semantic approach to analyze the explicit content [26].

Criteria of rigor and credibility for qualitative studies were followed: two of the authors performed triangulation and coding separately. Three authors discussed and suggested names defining the themes. Then, themes were discussed in the group of all authors. The group agreed on the final names (Table 1).

During this process, the main author gave name to sub-themes. The main- and sub-themes are presented in this paper and quotes are selected to illustrate the sub-themes.

### 2.6. Ethics

Regional Committee for Medical and Healthcare Ethics (REK no. 130447) and the Personal protection agency at Oslo University Hospital approved the study. The questionnaire was answered anonymously. Contact information was provided as a possibility to mail the research team in case of health care/psychological support.

## 3. Results

### 3.1. Quantitative Data for the Total Population

Table 2 provides sosiodemographic data for the total population responding to the CORONAPOP study.

The data in this section is not shown in Table 2, but refers to the total population. Some had been in quarantine or isolation (28.2%), 23.4% reported having risk factors for developing complications if infected, but few had been infected by COVID-19 (1.4%). More than half were generally concerned about the pandemic, while more than 80% were concerned for family and close friends. Concerns regarding current economy were reported by 21.8% and 25.3% had concerns in relation to economic loss. Nearly all (90.5%) reported that they had someone that would help if needed (social support).

### 3.2. Qualitative Analysis of the Open-Ended Question Related to Concerns

Comments were made by 1491 informants and they presented 1961 concerns in the open-ended question “Do you have other concerns related to the pandemic?” Analysis of sociodemographic differences between those who commented on concerns or not revealed that there were more respondents with higher education (86% vs. 83%, respectively, p = 0.022) commenting. None of the remaining variables were significantly different between the groups, but more women (34% vs. 30%, p = 0.050) commented on concerns.

### 3.3. Thematic Analysis

**Figure 2:**
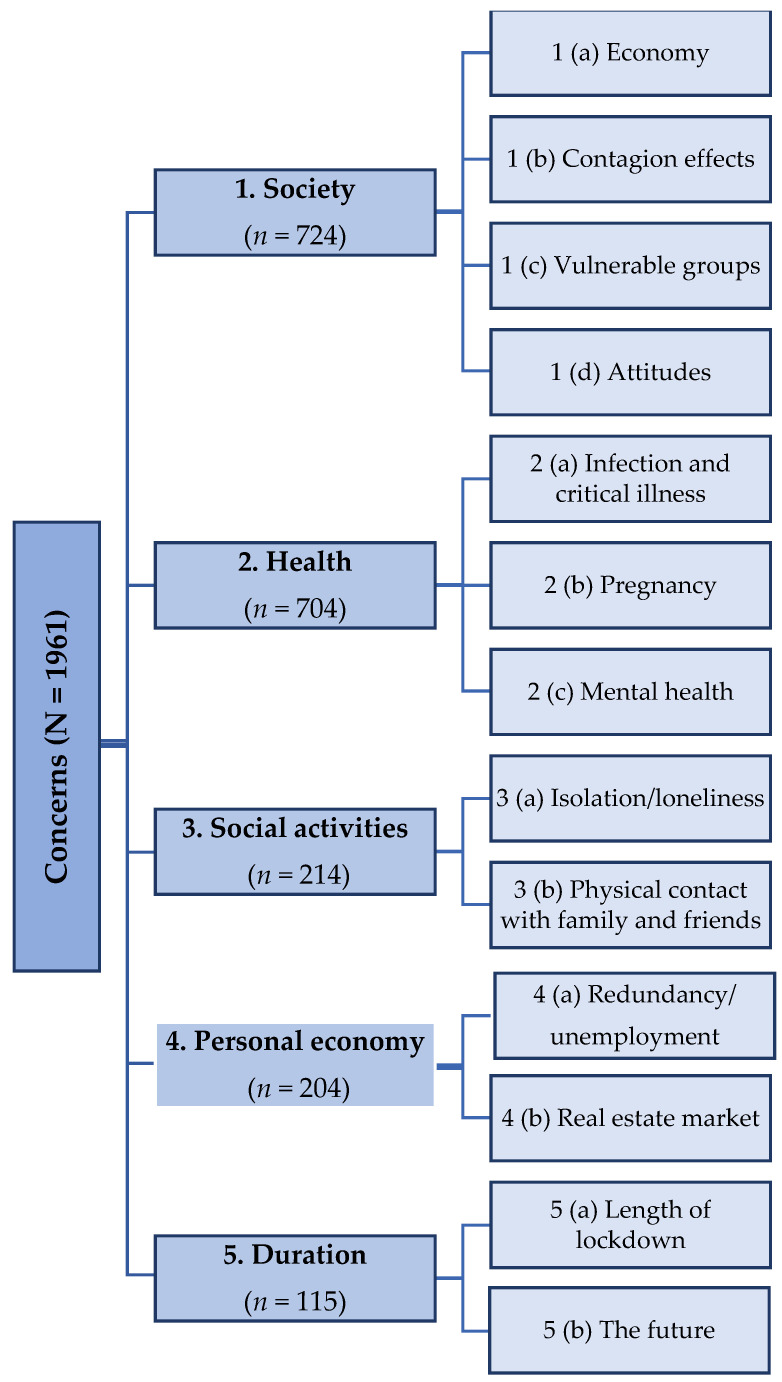
Themes and sub-themes from the qualitative analysis.

The thematic analysis identified five themes; society; health; social activity; personal economy and duration of the pandemic. For each theme, sub-themes appeared (Figure 2).

#### 3.3.1. Society

Seven hundred and twenty-four respondents commented on societal concerns. A wide range of topics appeared, including both national and global concerns. The highest proportion, though, were of a national character. Four sub-themes were identified in the thematic analysis: “economy”, “contagion effects”, “vulnerable groups” and “attitudes”.

##### Economy

Economic and societal consequences for the nation, both at short and long terms, were identified. This yields for the Norwegian welfare system including fear of collapse, but also bankruptcy in local companies. Some respondents were concerned about the global economy, poverty and the society but also world peace due to possible unstable political struggle following the pandemic. Some concerns were related to a less caring society in the future. The concerns yielded mostly in a global perspective, for poverty, refugees and the healthcare system in under-developed countries. Some addressed whether social distancing in Norway will remain and whether larger differences between people will appear. The following comments may shed light on these concerns:

…economic recession and unemployment and subsequently consequences: populism, unease, shortage of resources for other important issues…

…after-effects at a global perspective: distance between rich and poor may increase and those with limited resources will suffer most...

##### Contagion Effects

Fear that the capacity in hospitals would be congested (overloaded) leading to reduced treatment for patients with other diseases, both somatic and psychological, were some of the concerns. Lack of medication and personal protection equipment and an increase in people being infected were other concerns, but also contagiousness of the COVID-19 virus and the routes of infection. In addition, lack of knowledge due to the long-term consequences of the virus, including immunity and mutation and preparedness to meet the pandemic.

…consequences due to lack of knowledge on the routes of infection and why some become critically ill and other have mild/no symptoms…

…concerned for the healthcare system, both regarding the pandemic but also extended waiting lists in the after math...

##### Vulnerable Groups

Children, elderly and people with psychological/mental problems or drug abusers were examples of vulnerable groups, whether they receive needed treatment in the health care or social system. Most comments were related to children at risk during the lockdown, living in a family with violence or abuse, but even people with mental health issues.

…thinking of children who have been at home for several weeks with parents that abuse them, or are unable to take care of their children…

##### Attitudes

Some were concerned of other people’s indifference towards the health-authorities” and government’s advice and recommendations; 1–2 m distance, clean hands and stay at home if symptoms. Others wrote comments related to anxiety in friends and family members, or that the information was too negative and the restrictions too strict. Some of the comments were quite harsh.

…surprisingly many intelligent friends and family members that are “unable” to absorb the seriousness of the pandemic situation…

…people’s ignorance and stupidity. Young people who do not care…

…excessive hysteria…

#### 3.3.2. Health

We found 704 comments on personal health. Three sub-themes were identified: “infection and critical illness”, “pregnancy” and “mental health”.

##### Infection and Critical Illness

Many had concerns related to infecting family or friends in the risk groups, or colleagues and patients. More explicit, having a non-symptomatic COVID-19 infection and spreading the virus. Others were concerned about being infected themselves, especially when having risk factors. Most comments about critical illness if infected were related to elderly parents, children with chronic disease or functional impairment or family/friends with illness such as cancer, heart or lung disease. Some expressed uncertainty related to respiratory or other health conditions (pneumonia, blood clots, high blood pressure, born premature) if this puts one at risk. Only 22 revealed concerns related to the possibility of dying.

…being contagious and then infecting patients and my spouse, who is at risk…or…infecting patients at work…or…being the one to blame for putting colleagues in quarantine…

…it might be difficult to avoid the virus over a period. Concerned about being infected and dying, I am at risk…

##### Pregnancy

Many commented on concerns related to pregnancy especially being pregnant, giving birth without present partner, or their newborn child:

…giving birth alone, give birth during the pandemic situation, if my child gets sick and dies, loneliness during the maternity leave…

##### Mental Health

Psychological consequences such as increased level of anxiety, depression and eating disorders in already distressed persons were presented and some revealed rumination and concerns related to reduced psychological follow-up due to the pandemic. Other were concerned for the mental health of friends and family members.

…Loneliness and serious depression. It will be difficult to recover. Had depressions before the pandemic, which has become considerably worse. My migraine has also increased with more frequent seizures...

#### 3.3.3. Social Activities

Two hundred and fourteen comments on personal activities were reported. Two sub-themes were identified: “isolation/loneliness” and “physical contact with family and friends”.

##### Isolation/Loneliness

Impact of isolation on mental health, elderly or people living alone were concerns. In addition, social consequences when deprived of socialization with people outside one’s own household and development of negative relationships in the family. Missing physical contact was also part of loneliness. Young people expressed concerns related to missing opportunities and social life.

…sad that elderly family members must stay isolated. I am worried for them. They may die of other circumstances in this period and after staying alone their last days…

…concerns about regular life. I am young and feel that life disappears…This affects my mental health…

##### Physical Contact with Family and Friends

Not being able to visit or be together with family, friends and boy-/girlfriend was a concern, especially when they live in other parts of Norway than themselves or abroad, but also related to restricted social life in general.

…afraid I can’t see my love for a long time on a regular basis due to the restrictions on traveling. Long distance relationship…

…struggling—I can’t hang out with friends as usual. They are a huge comfort when I’m feeling blue…

#### 3.3.4. Personal Economy

The main theme society included concerns for national and global economy. In the present theme, we identified concerns related to personal economy, including 204 comments on consequences for self, family/friends or company.

Two sub-themes were identified: “redundancy/unemployment” and “real estate market”.

##### Redundancy/Unemployment

Most comments were related to unsecure work conditions; concerns of redundancy or losing one’s job, especially for people employed in private companies, art-related work and tourism. Others were concerned about not getting a job after completed studies.

…I fear that the economic recession and increased unemployment the COVID-19 virus have created will persist, and make it difficult to return to paid work…

##### Real Estate Market

In Norway, most people own their house/flat and economic consequences of the pandemic may influence the possibility of selling or buying if the price decrease or increase. The following comments reflect this:

…ripple effects for me and my husband’s economy—I hope we can keep the house after the pandemic...

…Selling price on our flat. Concerned of a decrease in value relative to what we payed...

#### 3.3.5. Duration

We found one hundred and fifteen comments on duration. Two sub-themes were identified: “length of lockdown” and “the future”.

##### Length of Lockdown

Most comments reflected on duration of the pandemic and the lockdown, often expressed with one word: duration. Others reflected on reduced possible to meet family and friends or effect on mental health.

…what if it never ends…

##### The Future

Many were concerned of a premature opening of schools and kindergartens and the future for children. Others expressed concerns over how society will develop after the pandemic, especially related to an economic and political crisis.

…worried that children and kindergarten employees are used as “guinea pigs” when society opens again and for vulnerable children. I believe this period will create great psychological distress for many, both children and adults…

…concerns related to the future—is this a glimpse of what lies ahead. The welfare state and the general economic situation in our society…

## 4. Discussion

Five main themes were identified and most comments were related to a broader concern for society and health. Empathy for others is prominent in the respondents in this study—for society (nationally and globally), but also concerns related to infecting others, and family members at risk for developing serious illness if infected, as seen in previous studies [8,18,20,21]. Concerns seem to be global and action should be taken to address these concerns since knowledge about concerns in the population is crucial for appropriate decision-making made by authorities in prevention and reduction of longer-term consequences of the pandemic. Targeted measures work best if they are in line with the public’s attitudes and concerns.

### 4.1. Society

For society, both national and global concerns were presented; on economy, for less fortunate people living in under-developed countries and unstable politics. A high proportion revealed concern for collapse of the national welfare system, companies going bankrupt but also concerns for sick people not receiving medical help due to crowded hospitals and even vulnerable groups not getting help.

As seen in the media, both in Norway and abroad, some believe that the COVID-19 situation is exaggerated; a politically induced problem and that people have unnecessary concerns. Modar [21] identified a similar concern. Some made harsh comments about this. Others were concerned of people’s indifference towards the health-authorities’ and government’s advice and recommendations leaving two groups with conflicting views. The proportion presenting hash comments was small in the present study, perhaps as a result of the transparent and joint communication from the Norwegian authorities (both government and health) establishing trust [6] and a strong team spirit [1]. People’s indifference may increase when the pandemic continues over time and people get exhausted from the restrictions.

### 4.2. Health

A larger proportion were more concerned of infecting family and friends, colleagues or patients than being infected themselves. Such concerns have also been related to symptoms of depression [16]. Flesia et al. [15] found higher distress in people living with others and in women, which may support our findings of concerns related to infecting family/friends, since people living alone are less prone to infect others. Others were concerned about a premature opening of kindergartens and schools and, thus, being close to a larger number of people and exposed to the virus. Some perceived this as an experiment. One of the recommendations from The International Task Force on Teachers for Education 2030 [27] is to “Prioritize teachers’ and learners’ health, safety and well-being” since they are exposed to COVID-19 virus at work and “have to deal with anxiety and stress”.

Some participants in the high-risk-group were concerned about getting critically ill and dying. Clotworthy et al. [20] found that some older or chronically ill people in Denmark felt overprotected and that their relatives were more concerned for them than they were themselves. A proportion of pregnant women in the present study expressed concerns related to giving birth or for the newborn child. Hessami et al.’s [28] systematic review and meta-analysis found a significant increase in anxiety among women during pregnancy and in the perinatal period, recommending support measures to be implemented. Perinatal distress was also found in a qualitative analysis of COVID-related comments in a leading online support forum for pre- through to post-birth in Australia [29].

### 4.3. Other Themes

There were substantially fewer comments related to the other themes, indicating that there may be less concerns about social activities, personal economy and duration of the pandemic at the early stage of the pandemic. This result may depend on the demographic characteristics of the sample, as the participants were highly educated and therefore, likely to earn a medium-high income. On the other hand, some concerns may have been included in social and health themes, but not expressed in words related to the latter themes. Christner et al. [30] found that empathy for loved ones was related to social distancing and highlighted considering social distancing as a moral behavior. The Norwegian authorities’ call to join a collective effort was an encouragement and thus, contributing to a strengthened team spirit and at the first outbreak few Norwegians were infected, hospitalized or deceased due to COVID-19 compared to other countries.

Regarding economic concerns, the Norwegian people are fortunate, living in a country with substantial economic resources. Most people in need may claim social benefits, in contrast to people living in the southern parts of Europe with less economic aid from the government [6]. Even if economic concerns were present in this study, they were not the most pressing concerns.

The themes about concerns in the present study are in line with the domains from WHO COVID-19 Snapshot Monitoring project (health, economic, emotional, work and future). They are also in line with most of the addressed concerns Sabat et al. [6] studied: losing a close person, becoming unemployed, health system becoming overloaded, closed school, small companies running out of business, recession and society getting more egoistic.

### 4.4. Methodological Considerations

#### 4.4.1. Strengths/Limitations

The study utilized questions with pre-categorized response options as well as open-ended questions to which respondents could elaborate. Some open-ended comments may have been influenced by the pre-categorized questions about concerns. On the other hand, we collected most information on the themes “society” and “health”, themes that were not specifically addressed in the quantitative part. In-depth interviews would have given a possibility to explore the concerns even further.

We collected data using social media with a snowballing effect. This resulted in a sample not stratified by gender, age or place of residence, with more women, younger people (few respondents over 59 years) and people living in urban areas and thus, not representative of the Norwegian population. A number of population-based studies performed at an early stage of the pandemic have collected data, using a web-based methodology. This technology allows for reaching many people but with limitations towards representativeness. Open-ended questions enabled us to reveal topics that were not known beforehand and these topics can be used in further research or future similar scenarios. The qualitative approach in the present study creates knowledge regarding concerns in the population and may create a basis for future recommendations from the authorities.

It may be considered a limitation that independent analysts were not consulted to ensure as much objectivity as possible in both the selection and coding of the textual analysis units. It may also be a limitation that we did not take into account whether people were pregnant; this point may be addressed in future studies.

#### 4.4.2. Implications for Public Health and Future Research

The study implies that concerns during the pandemic crisis are not limited to concerns about transmission, illness and death. Public health policies should consider that people have a variety of concerns during such circumstances.

An elaboration of the five main themes of concerns in In-depth interviews could identify other themes; in addition, the informants could suggest target measures for the authorities.

We recommend a further study with similar questions for the general population to study the impact of the pandemic restrictions over time. The study should preferably be stratified for gender, age and place of residence. The analysis should be blinded and inter-rater agreement levels calculated. Presence of physical or mental health problems prior to and during the pandemic should be included as confounding variables.

A future study could include specific questions and in-depth interviews with pregnant women and their partner.

## 5. Conclusions

Five themes were revealed in this qualitative part of the CORONAPOP study related to the first outbreak of the pandemic in Norway: society, health, social activity, personal economy and duration. Most comments were concerns with society and health. Empathy for others was prominent among the respondents in this study—for society (nationally and globally), but also concerns related to infecting others and family members at risk for developing serious illness if infected. The open-ended question elaborated more information on concerns than from a quantitative study.

## Figures and Tables

**Table 1:** Process of developing main themes.

Author 1	Author 2	Preliminary Themes	Final Theme Names
Societal Health related Psychological Vulnerable groups Worldwide consequences To early opening of the society Duration of the Pandemic	Worldwide consequences Societal Economic; personal, family, society, world The governmental handling of the pandemic; restrictions, to early opening of the society Social isolation, loneliness Mental health Vulnerable groups Contagious—health and death Duration of the pandemic The future—consequences of the pandemic	Medical concerns Sociological concerns Economic concerns Psychosocial concerns Concerns of duration	Society Health Social activities Personal economy Duration

**Table 2:** Sociodemographic data.

% (*n*)	Total Sample
**Respondents**	4527
**Gender %**	
Women	85.0 (3850)
Men	15.0 (659)
**Age groups %**	
18–29	25.5 (1156)
30–39	26.9 (1220)
40–49	20.6 (931)
50–59	16.9 (766)
60–69	7.8 (354)
70–79	1.8 (82)
≥80	0.4 (18)
**Place of residence %**	
Rural (<200 inhabitants)	4.4 (187)
Village (200–19,999 inhabitants)	25.2 (1141)
Town (20,000–99,999 inhabitants)	24.1 (1091)
City (≥100,000 inhabitants)	46.3 (2098)
**Education level %**	
higher (<12 years)	84.3 (3417)
lower (≥12 years)	15.5 (636)
**Employed or in education %**	
Before COVID-19 pandemic	87.8 (3971)
During COVID-19 pandemic	81.0 (3667)
**Civil status %**	
Married	60.0 (2716)
Boy/girlfriend	7.0 (318)
Widow	1.1 (48)
Divorced	4.1 (187)
Single	27.8 (1258)
**Living situation %**	
Living alone	22.0 (994)
With parents	7.0 (319)
Spouse	60.0 (2714)
Person > 18 years	16.1 (730)
Children < 18 years	34.2 (1547)

## Data Availability

The datasets used and/or analysed during the current study are available from the corresponding author on reasonable request.
